# In vitro dynamic model simulating the digestive tract of 6-month-old infants

**DOI:** 10.1371/journal.pone.0189807

**Published:** 2017-12-20

**Authors:** Francesca Passannanti, Federica Nigro, Marianna Gallo, Fabio Tornatore, Annalisa Frasso, Giulia Saccone, Andrea Budelli, Maria V. Barone, Roberto Nigro

**Affiliations:** 1 DICMAPI, University of Naples Federico II, Naples, Italy; 2 University of Rome Niccolò Cusano, Engineering Department, Rome, Italy; 3 Heinz Innovation Center, Nijmegen, Netherlands; 4 Department of Translational Medical Science, University of Naples Federico II, Naples, Italy; 5 European Laboratory for the Investigation of Food Induced Disease (ELFID), Naples, Italy; University of Insubria, ITALY

## Abstract

**Background:**

In vivo assays cannot always be conducted because of ethical reasons, technical constraints or costs, but a better understanding of the digestive process, especially in infants, could be of great help in preventing food-related pathologies and in developing new formulas with health benefits. In this context, in vitro dynamic systems to simulate human digestion and, in particular, infant digestion could become increasingly valuable.

**Objective:**

To simulate the digestive process through the use of a dynamic model of the infant gastroenteric apparatus to study the digestibility of starch-based infant foods.

**Design:**

Using M.I.D.A (Model of an Infant Digestive Apparatus), the oral, gastric and intestinal digestibility of two starch-based products were measured: 1) rice starch mixed with distilled water and treated using two different sterilization methods (the classical method with a holding temperature of 121°C for 37 min and the HTST method with a holding temperature of 137°C for 70 sec) and 2) a rice cream with (premium product) or without (basic product) an aliquot of rice flour fermented by *Lactobacillus paracasei* CBA L74. After the digestion the foods were analyzed for the starch concentration, the amount of D-glucose released and the percentage of hydrolyzed starch.

**Results:**

An in vitro dynamic system, which was referred to as M.I.D.A., was obtained. Using this system, the starch digestion occurred only during the oral and intestinal phase, as expected. The D-glucose released during the intestinal phase was different between the classical and HTST methods (0.795 grams for the HTST versus 0.512 for the classical product). The same analysis was performed for the basic and premium products. In this case, the premium product had a significant difference in terms of the starch hydrolysis percentage during the entire process.

**Conclusions:**

The M.I.D.A. system was able to digest simple starches and a more complex food in the correct compartments. In this study, better digestibility of the premium product was revealed.

## Introduction

With the current increase in the prevalence of food-related pathologies (type 2 diabetes, cardiovascular diseases and food allergies) [1–2], there is a growing interest in determining the effect of food on human health. Digestion is, therefore, a key step in delivering compounds that will have beneficial or deleterious effect on human health. Carbohydrates are a good example of such compounds. They are extremely important during the first phases of the infant nutrition, and their digestion, in food in general and in baby foods in particular, has not been properly investigated. Most of the carbohydrates in the diet are composed of starch [3]. The first stage of starch digestion takes place in the mouth where saliva acts to start to break down carbohydrates due to an enzyme called α-amylase, but its activity lasts for a relatively short time because α-amylase is inactivated by the gastric acid when the bolus reaches the stomach. The digestion process continues in the stomach and finally in the intestine, where pancreatic enzymes help to break down fats, proteins and carbohydrates [4]. Much of the starch gets digested in the small intestine by the action of pancreatic α-amylase, which hydrolyzes starch mainly to α-limit dextrins [5]. Intestinal brush border enzymes further digest these products to monosaccharides. The most important brush border enzymes are limit dextrinase and glucoamylase, which act on oligosaccharides composed of more than three simple sugars, and maltase, sucrase, and lactase which then hydrolyze maltose, sucrose and lactose, respectively, into their constituent monosaccharides. Understanding the mechanisms of infant formula disintegration, in the infant gastrointestinal tract is a key step for developing new formulas with health benefits for the neonate. Human clinical trials remain undoubtedly the best approach to study food digestion [6]. However, in vivo assays cannot always be conducted because of ethical reasons, technical constraints and cost. To bypass those drawbacks, an alternative way to study digestion is to use in vitro systems [7]. In order to get closer to the physiological reality of the gastrointestinal tract, in vitro dynamic digestion systems have been developed [8]. These models, able to simulate the digestive process both of an adult and a child, include regulation of the pH, dynamic flows of food and concentration of dygestive enzymes in the different compartments [7]. In vitro digestion methods are ethically superior, faster and less expensive than in vivo techniques, and provide a useful alternative for a fast screening of food digestion [9].

The aim of the present study was to develop a dynamic *in vitro* digestive system based on infant physiology including the oral, gastric and small intestinal phases of digestion, to estimate the starch digestibility of two different categories of baby foods: rice starch-based foods and rice cream-based foods.

## Materials and methods

### Chemicals

All materials were standard analytical grade. α-amylase was obtained from *Bacillus licheniformis* (E-BLAAM EC.3.2.1.1; Megazyme International Ireland Ltd., Wicklow, Ireland) and was supplied at a concentration of 10000 U/ml on soluble starch at pH 6.5 and 40°C. Glucoamylase was obtained from *Aspergillus niger* (AMG Conc. BG; Novozymes, Bagsværd, Denmark) and had an activity of 1300 AGU/g. Pepsin (P6887; Sigma-Aldrich S.r.l. Milan, Italy) was obtained from porcine gastric mucosa and had an activity between 3200 and 4500 U/mg protein. Creon® 10000 U.Ph.Eur. (Abbott Products S.p.A; Sesto San Giovanni (Mi), Italy), which is normally used in the treatment of pancreatic exocrine insufficiency, was available as brown/clear capsules. Each capsule contained lipase (10000 Ph.Eur. units), amylase (8000 Ph.Eur. units), and protease (600 Ph.Eur. units). CaCl_2_, KCl, KH_2_PO_4_, NaHCO_3_, NaCl, MgCl_2_(H_2_O)_6_, (NH_4_)_2_CO_3_ and HCl were purchased from Sigma-Aldrich (Sigma-Aldrich, Sigma-Aldrich S.r.l. Milan, Italy).

### Rice starch: Classical and HTST methods

Rice starch (lot number: 1221660205; product code: 10051400) was provided by Heinz Italia S.p.A, Latina, Italy. Water and rice starch were selected to outsource the fine tuning of the M.I.D.A. The volume selected was 45 ml, and the mass percentage of starch contained in it was 5.5% w/v, which corresponds to the average starch content reported on the nutritional label of different baby foods. This mixture of rice starch and water was then autoclaved in two different ways to simulate the main sterilization techniques for baby foods: the classical sterilization technique (classical) (holding temperature: 121°C, holding time: 37 min) and High-Temperature-Short-Time (HTST) (holding temperature: 137°C, holding time: 70 sec).

Both sterilization treatments were carried out in a lab autoclave (Falc Instruments, ATV 80), to reproduce the stabilization processes generally used for low acid products.

### Rice cream: Fermented (premium) or not (basic)

The rice cream-based foods were both made up of rice cream (provided by Heinz Italia S.p.A., Latina, Italy; lot number: 4277) and mineral water. In particular we used mineral water and rice cream (13.64% w/v), based on a recipe recommended for six-month-old infants, which corresponded to approximately 3 spoons of rice cream with 180 ml of water added, as suggested by the label of common commercial products, fermented or not fermented. For these analyses proteolytic enzymes were also added to the Simulated Gastric Fluids (SGF). The first food was a commercial rice cream, which was referred to as the “basic product”, and the second food was the same product with an 15% w/w addition of rice cream fermented by *Lactobacillu*s *paracasei* CBA L74, which was referred to as the “premium product”. The fermentation process has been previously described [10]. Briefly, the fermented cereals were prepared from cereal flours fermented by *Lactobacillus paracasei* CBA L74 (Heinz Italia SpA), International Depository Accession Number LMG P-24778. The fermentation was started in the presence of 10^6^ bacteria, achieving 10^8^ CFU/ml after a 20 h of incubation at 37°C. After heating at 85°C for 20 sec, to inactivate the live bacteria, the fermented cereal was spray dried (target outlet temperature: 85°C), and a 15% w/w aliquot was added to the rice cream to obtain the premium product. The final fermented cereal powders contained bacterial bodies and fermentation products.

### Simulated Digestive Fluids

The Simulated Digestive Fluids were the following: Simulated Salivary Fluid (SSF), Simulated Gastric Fluid (SGF) and Simulated Intestinal Fluid (SIF). They were made up of distilled water, enzymes, CaCl_2_ and the corresponding electrolyte stock solutions, whose composition is given in Table 1.

**Table 1:** The chemical composition of the electrolyte stock solutions contained in the Simulated Digestive Fluids, used in M.I.D.A.

*Electrolyte stock solution*	SSF (pH = 7)	SGF (pH = 3)	SIF (pH = 7)
	ml	ml	ml
**KCl 0.5 M**	15.1	6.9	6.8
**KH**_**2**_**PO**_**4**_ **0.5 M**	3.7	0.9	0.8
**NaHCO**_**3**_ **1 M**	6.8	7.2	5.26
**NaCl 2 M**	-	11.8	9.6
**MgCl**_**2**_**(H**_**2**_**O)**_**6**_ **0.15 M**	0.5	0.4	1.1
**(NH**_**4**_**)**_**2**_**CO**_**3**_ **0,5 M**	0.06	0.5	-
**HCl 6 M**	0.49	1.3	0.7
**H**_**2**_**O**	373.35	371	375.74

Distilled water was added to bring the volume of the solutions to 400 ml (adapted from [11]). SSF: Simulated Salivary Fluid; SGF: Simulated Gastric Fluid; SIF: Simulated Intestinal Fluid.

As suggested by Minekus M et al [11], the Simulated Digestive Fluid in each compartment was mixed with an equal volume of the fluid coming from the previous compartment to achieve a ratio of 50:50 (v/v) during digestion e.g., since the volume of each test food was 45 ml, 45 ml of SSF, 80 ml of SGF (instead of 90 ml as 10 ml of bolus was sampled for analysis) and 80 ml of SIF (instead of 160 ml as half of the gastric content was sampled for analysis) were prepared for the *in vitro* digestion procedure. The compositions of the simulated digestive fluids for the *in vitro* digestion of the rice starch-based foods and the rice cream-based foods are shown in Table 2. Since the initial pH value of every food was different, the amount of 1 M HCl and 1 M NaHCO_3_ required to adjust the pH to the proper value in each digestive compartment was determined in a test prior to digestion and then added to each simulated digestive fluid. In particular, the gastric pH choosen was near 3, because of the gradual reduction observed from the first month (pH value 6–7) to the second year of life (pH 1–2) [12]. In the absence of information of the exact pH value near the sixth month of life, an intermediate value was choosed.

**Table 2:** The chemical composition of the Digestive Simulated Fluids used in the M.I.D.A. for *in vitro* digestion of two rice starch-based foods (adapted from [11]).

*in vitro* digestion of	Chemicals	SSF	SGF	SIF
rice starch-based foods	**Electrolyte stock solution**	31.5 ml of SSF	60 ml of SGF	44 ml of SIF
	**Enzymes**	0.675 ml of α-amylase (75 U/ml)	-	2.73 ml of α-amylase (170.5 U/ml)
		-	-	2.308 g of glucoamylase (0.11*∙* pancreatic α-amylase activity)
	**CaCl**_**2**_ **0.3 M**	0.225 ml	0.04 ml	0.16 ml
	**HCl 1 M**	0.2 ml	0.22 ml	-
	**Distilled water**	to bring up to volume	to bring up to volume	to bring up to volume
rice cream-based foods	**Electrolyte stock solution**	31.5 ml of SSF	60 ml of SGF	44 ml of SIF
	**Enzymes**	0.675 ml of α-amylase (75 U/ml)	47.33 mg of pepsin (1000 U/ml)	115.425 mg of pancrelipase
		-	-	0.52 g of glucoamylase (0.11*∙* pancreatic α-amylase activity)
	**CaCl**_**2**_ **0.3 M**	0.225 ml	0.04 ml	0.16 ml
	**HCl 1 M**	0.04 ml (basic product)	1.945 ml (basic product)	- (basic product)
		0.02 ml (premium product)	1.33 ml (premium product)	0.02 ml (premium product)
	**NaHCO**_**3**_ **1 M**	-	-	0.18 ml (basic product)
		-	-	0.02 ml (premium product)
	**Distilled water**	to bring up to volume	to bring up to volume	to bring up to volume

### Structure and dimensions of the *in vitro* dynamic digestive system M.I.D.A

**Fig 1:**
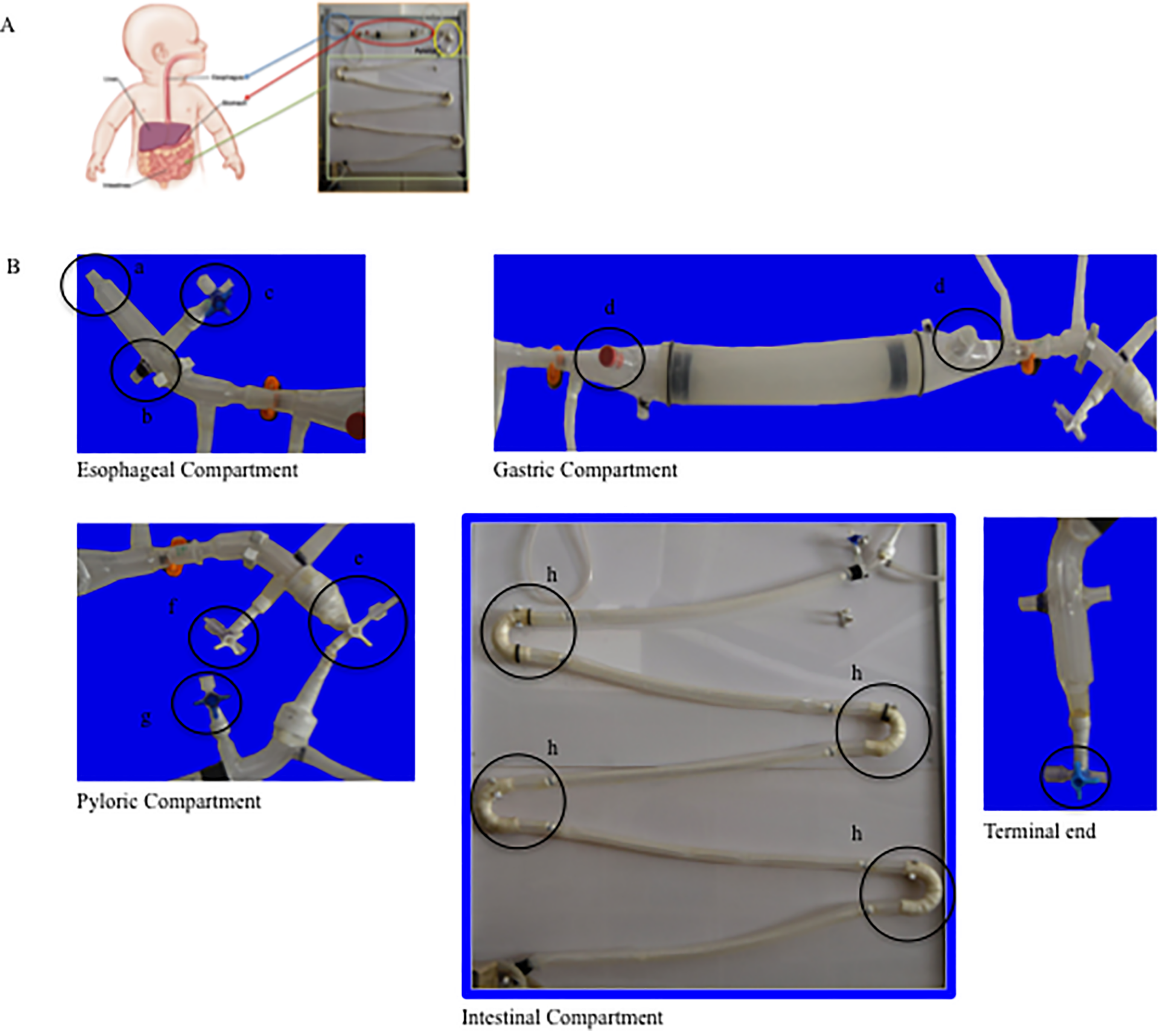
The M.I.D.A. *in vitro* dynamic digestive system. A) the completed system; B) M.I.D.A. esophageal compartment (a: injection point for the bolus; b: connection hole with the 37°C water bath; c: three-way valve for the input of the Simulated Gastric Fluid), gastric compartment (d: injection points for the glass*-*electrode of the pH Meter to monitor the pH of the stomach chyme.), pyloric compartment (e: three-way valve simulating pylorus; f: three-way valve for sampling gastric content; g: three-way valve for the input Simulated Intestinal Fluid), intestinal compartment (h: four connecting loops), and the terminal end of the intestinal compartment (i: three-way valve for chyle sampling).

An *in vitro* dynamic digestive system, referred to as the M.I.D.A. was designed for this study reproducing the physiology of the gastrointestinal tract of a six-month-old infant. It consisted of four consecutive compartments simulating the esophagus, the stomach, the pyloric sphincter and the intestine as shown in **Fig 1A**.

These compartments (except for the pyloric sphincter) were composed of two concentric tubes. Food and the digestive juices flowed in the inner tube, while the annular space between the concentric tubes allowed the continuous circulation of water at 37°C, which was pumped from a thermostatic bath to maintain the normal human body temperature in each compartment.

The esophageal compartment, shown in **Fig 1B**, was made entirely of Pyrex and had an inner diameter of 10 mm and a lenght of 90 mm. In the terminal area, the tube had a curvature and was connected to the next compartment by means of conically tapered ground glass joints. The gastric compartment was made up of two conical glass elements and two white concentric silicon rubber tubes, as illustrated in Fig 1B, which made it easy to handle to mimic physiological peristalsis motions.

The inner tube of the stomach compartment had an inner diameter of 35 mm, a wall thickness of 1 mm and a length of 185 mm. The outer tube of the stomach compartment had an inner diameter of 40 mm, a wall thickness of 2 mm and a length of 210 mm. The global length of the stomach, including the conical glass elements, was 385 mm, while the overall volume of the gastric compartment was approximately 280 ml, as the capacity of stomach in infants varies between 90 and 500 ml [13]. The pyloric compartment, the small intestine and the terminal end are also shown in Fig 1B. The compartment simulating the small intestine consisted of five tubular sections, each of which was formed by two white concentric silicon rubber tubes and connected to each other using four loops. The inner tube of the intestinal compartment had an inner diameter of 10 mm and a wall thickness of 1 mm. The outer tube had an inner diameter and a wall thickness of 20 and 1 mm, respectively. During the small intestinal digestion, these tubes were continuously squeezed to reproduce the peristaltic and segmentation motions. The global length of the intestinal compartment was 350 cm. These dimensions were selected based on the final chyle volume (160 ml) and on the average length of the small intestine in infants (380 cm, [14]). The loops were made of ABS (acrylonitrile-butadiene-styrene), and on the inside, smaller glass loops were added to create the gap necessary to allow water circulation from the thermostatic bath. The small intestinal compartment ended with two concentric glass tubes (inner diameter of 10 mm) attached to a three-way valve whereby it was possible to collect samples of the small intestinal contents.

### *In vitro* digestion protocol

The experimental *in vitro* digestion protocol was developed based on the consensus paper from INFOGEST “*A standardised static in vitro digestion method suitable for food–an international consensus*” [11].

The only enzyme having physiological significance in the oral phase was α-amylase, and its activity in this work corresponded to the value suggested for adults by the consensus paper from INFOGEST because of the similar levels between salivary α-amylase in the adult and in 6-month-old-infants [15]. The bolus had a pH value of 7 as recommended in the previous work, since it was the average value detected among neonates [16] and children [17]. Both the rice starch-based foods and the rice cream-based foods were liquid, and mastication was therefore not required. The M.I.D.A. system allowed the measurement of the starch content at several time points starting from time point 0 (T0) up to 248 min (T248). To assess the amount of digested starch, samples were collected every fifteen min from the oral phase (T2) up to 242 min (T242). The last samples (T242- T248) were collected at closer time points to monitor the emptying of the M.I.D.A. system. In the first stage of the *in vitro* digestion, 45 ml of the food was split into nine sterile 10-ml Falcon tubes (each of which contained 5 ml of food, corresponding to the volume of a baby feeding-spoon). Then, in turn, 5 ml of the SSF was added to each Falcon tube, and the food-SSF mixture was placed in an incubator at 37°C for 2 min simulating the oral compartment. After the oral phase, one of the nine boluses was immediately frozen at -20°C and stored for successive analysis. The other boluses went through gastric digestion.

After incubation, every two minutes, 10 ml of bolus was inserted into the esophageal compartment together with 10 ml of SGF. Porcine pepsin was added to the SGF during gastric digestion of the rice cream-based foods only and was not required for rice starch-based foods because of the low protein content in isolated rice starch [18]. The enzymatic activity used was 1000 U/ml of gastric contents as suggested by Tavares AMP [19]. The gastric digestion lasted 2 hours, and eight samples were collected every 15 minutes to measure the starch and D-glucose content during this phase. Between each sampling, the stomach was regularly gently hand-squeezed, to mix the contents. All samples were kept frozen at -20°C until analysis. Once the gastric digestion was completed, the gastric emptying time was evaluated using a power exponential mathematical Eq (1), as suggested by Elashoff JD, et al [20]:

$${y(t)=2^{-(\frac{t}{T/2})}}^{\beta }$$ Eq (1)

where *y(t)* represents the fraction of the chyme remaining in the stomach at time *t; β* is the coefficient describing the shape of the curve; *T*/2 represents the gastric emptying half time calculated using Eq 2 [21]:

$$T/2=V_{0}∙(0.1797-0.1670^{-K})$$ Eq (2)

*T*/2 depends on V_0_, which is the volume of food going through digestion, and on *K*, which is the caloric density of the foods. The value of *T*/2 was 2.14 min for the rice-starch based foods and 3.17 min for the rice cream-based foods. The value of *β* was fixed to 1.5 according to Havenaar R, et al [22]. The gastric emptying time was 20 min for the rice starch based-foods and 28 min for the rice cream-based foods. In addition, when the gastric emptying begins, the pyloric sphincter opens briefly and closes approximately four times a minute in infants [23] to allow small volumes of chyme to pass through to the duodenum. These volumes were calculated using Eq 1 and are presented in Table 3.

**Table 3:** The volumes of chyme delivered into the duodenum every 15 seconds in the M.I.D.A. gastric emptying.

Rice starch-based foods		Rice cream-based foods	
Δt (min)	Volume of chyme (ml)	Δt (min)	Volume of chyme (ml)
0–2	6.22	0–3	4.2
2–4	2.36	3–6	1.56
4–8	0.608	6–12	0.4
8–20	0.034	12–28	0.024

During intestinal digestion, the volumes of chyme reported in Table 3 were input into the small intestinal compartment along with identical volumes of SIF, to achieve the final ratio of 50:50 (v/v). Analogous to the gastric phase, the duration of the intestinal digestion was 2 hours, and samples were taken every 15 minutes; thereafter, 10 ml of the residual intestinal content was collected every 2 minutes until completion. Also in this case, the small intestine compartment was gently hand-squeezed to mix the contents. Globally, 11 samples were obtained during intestinal digestion and were then stored frozen until analysis. Data on the pH of the small intestine in healthy infants was found to be lacking in the literature [12], but intestinal pH has been recorded to be similar between adults and children [24] and ranges from 6 and 7.5 [25]. A value of 6 was selected as it represented an optimal value for both α-amylase and glucoamylase. For reasons of simplicity and cost, the pancreatic enzymes (lipases, proteases and amylase) were added to the digestion mixture in the form of pancrelipase (CREON® 10000), which is a porcine pancreatic extract containing multiple enzyme classes [25]. As recommended by the Cystic Fibrosis Foundation [26], 1000 lipase units per kilogram per meal were used. The mean weight in infants at 6 months (evaluated using the World Health Organization (WHO) weight-for-age charts) was 7.695 kg corresponding to a dose of 7695 lipase units, 770 protease units and 6156 amylase units per meal. Creon 10000 was not necessary for digestion of rice starch-based foods because of their low lipid and protein content; therefore, the intestinal enzymatic digestion was simply carried by α-amylase using an amount corresponding to a duodenal activity of 170.5 U/ml [18]. The amount of glucoamylase added to the SIF was defined by a previous work [27] in which a ratio of glucoamylase activity to pancreatic α-amylase activity of 0.11 was discovered in adults and children’s duodenal aspirates.

### Chemical analyses and calculations

Twenty-one samples were collected during each experiment. The starch content was quantified enzymatically (AOAC Method 996.1, AACC Method 76–13.01) using the Megazyme Total Starch Assay Kit (Cat. No. K-TSTA 09/14, Megazyme, Wicklow, Ireland). Samples were filtered through Whatman No. 1 filter paper; then, 2 ml was taken and placed into 15 ml glass test tubes along with 8 ml of 95% v/v ethanol. These solutions were mixed vigorously on a vortex mixer and, after standing at room temperature for 30 min, were centrifuged at 1800 g for 10 min. The supernatant solutions were decanted, and starch was redissolved in 1 ml of water using a boiling water bath to aid redissolution. The volumes were adjusted to 3.9 ml with 100 mM acetate buffer at pH 5.0. Thereafter, 0.1 ml of AMG diluted 50-fold was added, and the solutions were incubated in a water bath at 50°C for 30 min. Aliquots of 0.1 ml of the diluted solutions were transferred to glass test tubes, and 3 ml of the GOPOD reagent was then added. After incubating the tubes at 50°C for 20 min, the absorbances for each sample were read against a blank at 510 nm.

D-glucose was measured using the Megazyme D-glucose Assay Kit (K-GLUC 09/14, GOPOD format, Megazyme, Wicklow, Ireland): 3 ml of GOPOD reagent was added to 0.1 ml of samples containing D-glucose and incubated at 40–50°C for 20 min. Absorbances were read at 510 nm against the blank. Digestion experiments were performed in triplicate for each food. The results are represented as the mean grams of starch and D-glucose in each sample as a function of the time of sampling.

The degree of conversion of starch was calculated using Eq 3:

$$x_{starch}=\left(\frac{g_{starch}-g_{starch(t)}}{g_{starch(t=0)}}\right)\%$$ Eq (3)

The D-glucose released (g) was calculated by subtracting the amount of D-glucose at t = 0 min (after preparing the foods and before the oral digestion) from the amount of D-glucose detected in each sample collected at time *t* according to Eq (4):

D−glucosereleased(t)=[D−glucose(t)]−[D−glucose(t=0)] Eq (4)

The amount of starch digested into D-glucose at each time of sampling *t* was calculated using the Eq 5 [28]:

$$\%SH(t)=\frac{S_{h}(t)}{S_{i}}=\frac{0.9∙G_{R}(t)}{S_{i}}$$ Eq (5)

where *%SH* is the percentage of starch hydrolysis at the sampling time *t*; *S*_*h*_ is the amount of starch hydrolized to D-glucose (g); *0*.*9* represents stoichiometric constant of D-glucose content conversion into starch; *G*_*R*_ is the amount of released D-glucose calculated using Eq (4); and *S*_*i*_ is the initial amount of starch (g) evaluated differently according to the tested foods. For the starch-based foods, *S*_*i*_ is the starch content measured before the sterilization treatments. For the rice cream-based foods, *S*_*i*_ is the starch content measured after dissolving the rice cream into water.

### Statistical analysis

Student t-test was used. Each test was conducted in triplicate. GraphPad Prism version 7.0 (GraphPad Software, Inc.) was used for the statistical analysis. Assuming no difference (the null hypothesis), the significance was determined using the *p* value. Differences were considered statistically significant if there was a probability greater than or equal 95% to refuse the null hypothesis.

## Results

### Starch digestion analysis after classical and HTST sterilization in the M.I.D.A. system

**Fig 2:**
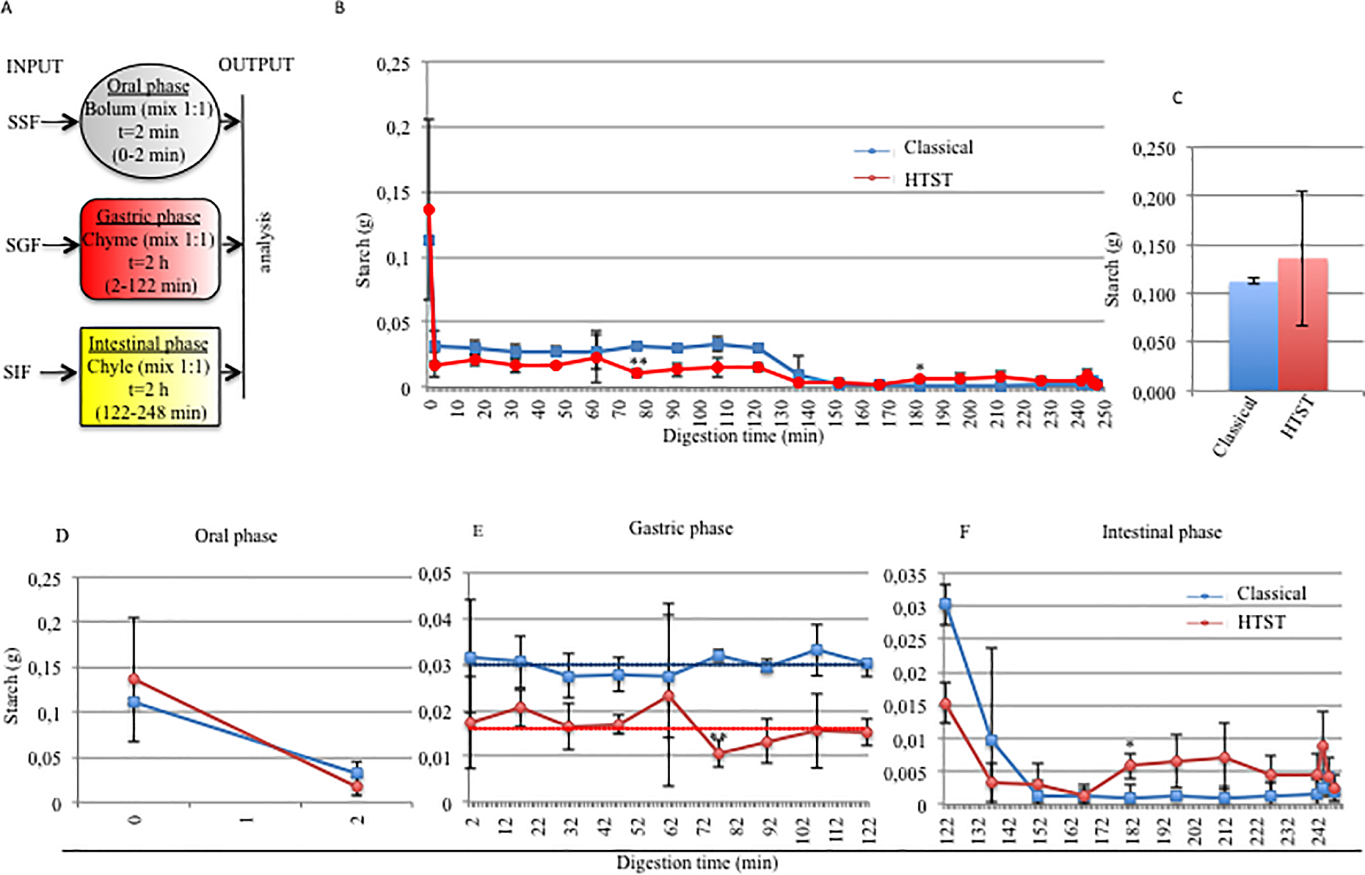
Starch digestion in the M.I.D.A. system of rice starch treated with the classical and HTST methods. A) The model describes a three-step procedure simulating the digestive processes in the mouth, stomach and intestine through the use of Simulated Digestive Fluids and physiological transit time: SSF (Simulated Salivary Fluid), SGF (Simulated Gastric Fluid), SIF (Simulated Intestinal Fluid). B) Effect of the sterilization method on the starch content during the entire digestion process (blue: classical method; red: HTST). C) Comparison of the starch content at 0 min between the food sterilized using the classical method (blue) and the food sterilized using the HTST method (red). Comparison of the starch content during the oral phase (D), the gastric phase (E) and the intestinal phase (F). In E, the blue dotted line represents the mean starch content observed during the entire gastric phase for the starch treated with the classical sterilization method, while the red dotted line for the starch treated with the HTST method. Each test was conducted in triplicate. Dots and squares represent the mean values, and bars represent the standard deviation. Student’s *t*-test * = *p*<0.05, ** = *p*<0.01.

The *in vitro* digestion process is shown schematically in **Fig 2A.** It was comprised of three phases replicating the oral, gastric and intestinal phases of digestion *in vivo*.

**Fig 2** shows an overall view of the digestion times (**B**), the starch level at T0 (**C**) and the separate steps of the digestion (**D, E, F**). The different starch content during the oral phase (from 0 min to 2 min) in the rice starch-based foods (classical and HTST) is reproduced in Fig 2D. After 2 min of incubation at 37°C with α-amylase, 0.032 g of starch was present in the food sterilized using the classical method and 0.017 g in the food sterilized using the HTST method. This difference was not statistically significant, suggesting that the different heat treatmements did not alter the oral digestibility. To obtain a better understanding, the degree of starch conversion was calculated using equation number 3, described in the methods section. The degree of conversion of starch was 71.43% for the food sterilized using the classical method and 87.5% for the food sterilized using the HTST method, demonstrating that most of the starch digestion took place in the oral compartment. The development of the gastric starch digestion (from 2 min to 122 min) in the rice starch based-foods (classical and HTST) is shown in Fig 2E. During the entire gastric phase, the starch content of the HTST (mean starch content: 0.016 g) was less than the classical product (mean starch content: 0.03 g); however, also in this case the difference was not statistically significant. Interestingly, in both cases, only a fluctuating trend around the mean value was observed, confirming that no starch digestion occurred during the gastric phase in the M.I.D.A. system.

The evolution of the starch content for both classical and HTST methods during the last stage of *in vitro* digestion (from 122 min to 248 min) is described in Fig 2F, and this shows that the starch was completely digested in this compartment. In conclusion, regardless of the sterilization method used, the digestion was almost complete after 150 and 170 min but not before. This time corresponded to the intestinal phase digestion when the Simulated Intestinal Fluid (SIF), which contained α-amylase and glucoamylase, was introduced in the M.I.D.A. system. The results achieved demonstrated that the M.I.D.A dynamic digestive system could digest starch in the right compartment and allowed punctual monitoring of the digestion process.

### Glucose and starch hydrolysis analysis after classical and HTST sterilization in the M.I.D.A. system

**Fig 3:**
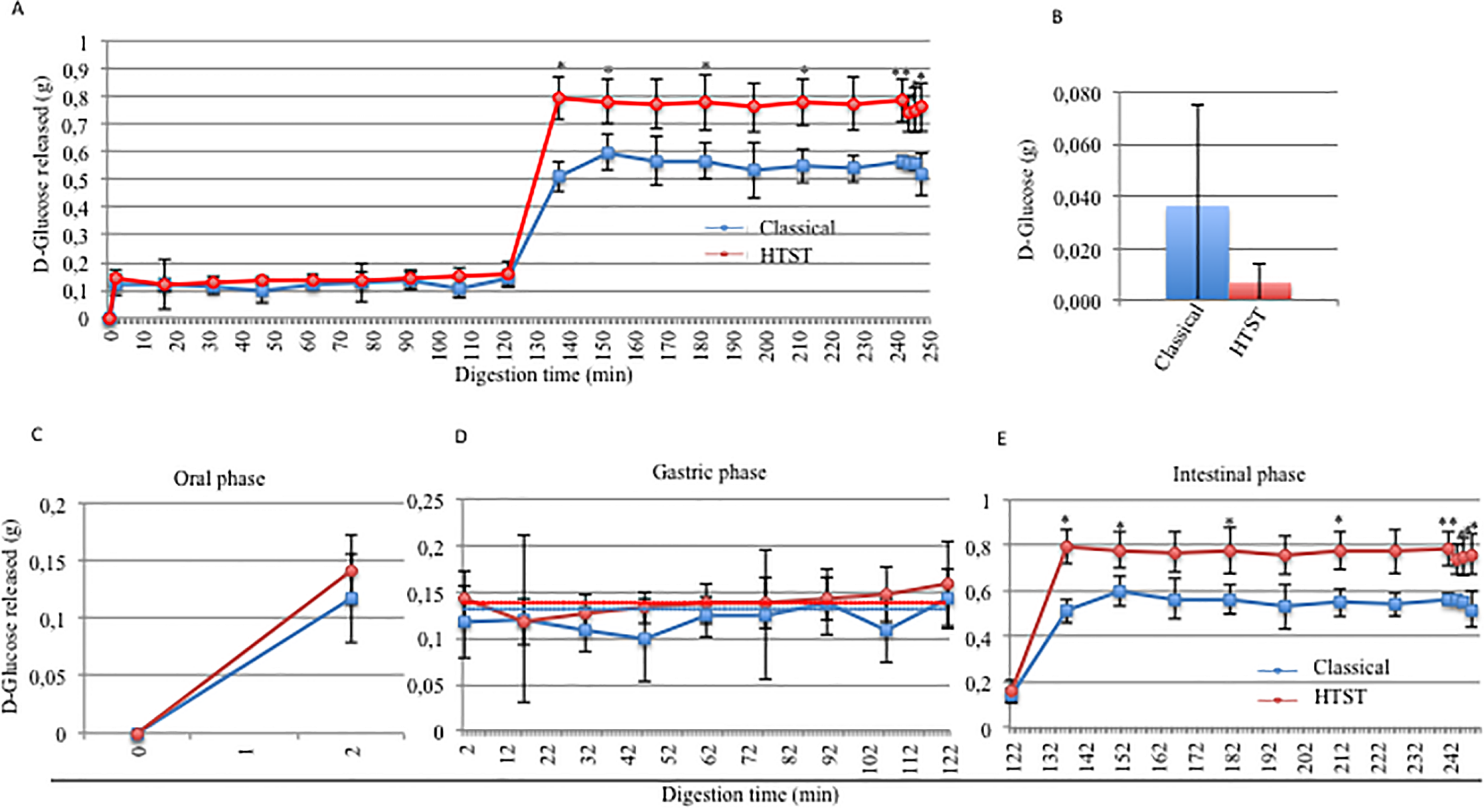
Effect of the sterilization method on the release of D-glucose. A) D-glucose released during the entire digestion process. B) D-glucose found at T0; D-glucose released during the oral (C), the gastric (D) and the intestinal steps (E). (blue: classical method; red: HTST). In D, the blue dotted line represents the mean D-glucose content observed during the entire gastric phase for starch treated using the classical sterilization method, while the red dotted line represents the mean D-glucose content for starch treated using the HTST method.

To better monitor the starch digestion in the M.I.D.A. system, we performed two different analyses of the samples obtained: the amount of D-glucose released and the percentage of starch hydrolysis. The D-glucose released was calculated using Eq 4, described in the methods section. In **Fig 3,** the amount of D-glucose released both during the entire digestion (**A**), at T0 (**B**) and during each separate step (**C, D, E**) is shown. At the time 0 point (Fig 3B), it was possible to note that the sterilization treatment appeared to affect the levels of D-glucose present at 0 min. In fact, a reduction in the glucose levels in the HTST (0.007 g) compared to the classical product (0.036 g) was present, but this difference was not statistically significant. D-glucose was released during only the oral (Fig 3C) and intestinal phase (Fig 3E), as expected. Indeed, after the first 15 min of the intestinal phase, there was 0.512 g of D-glucose in the food sterilized using the classical method with an increase of 72.07%, and 0.795 g of D-glucose in the food sterilized using the HTST method with an increase of 80%. In the later steps (from 137 min (T137) to 246 min (T248)), there were no significant changes in the amount of D-glucose released. During the intestinal phase of the digestion process (from 122 min to 248 min), there was a statistically significant difference between the two sterilization methods suggesting that the different heat treatments could alter the intestinal release of D-glucose from starch. These data confirmed that the M.I.D.A. system could digest starch in the correct compartments.

**Fig 4:**
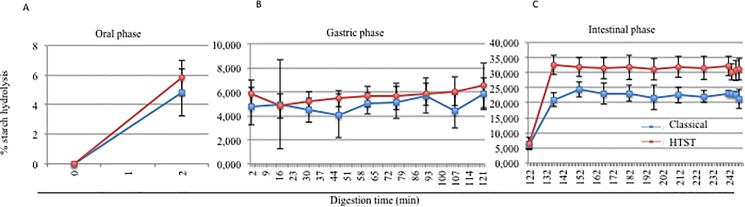
Effect of the sterilization method on the starch hydrolysis. Percentage of starch hydrolysis during the oral (A), gastric (B) and intestinal phases (C). (blue: classical method; red: HTST). Each test was conducted in triplicate. Dots and squares represent the mean values, and bars represent the standard deviation. Student’s *t*-test * = *p*<0.05, ** = *p*<0.01.

For a better understanding of the starch conversion into D-glucose, the percentage of starch hydrolysis was analyzed using the Eq 5, described in the methods section. **Fig 4**shows the percentage of starch hydrolysis both during the oral (**A**), gastric (**B**) and intestinal steps (**C**). Starch hydrolysis increased during the oral (Fig 4A) and intestinal phases (Fig 4C).

### Starch analysis of rice cream-based foods with and without fermentation digested in the M.I.D.A. system

**Fig 5:**
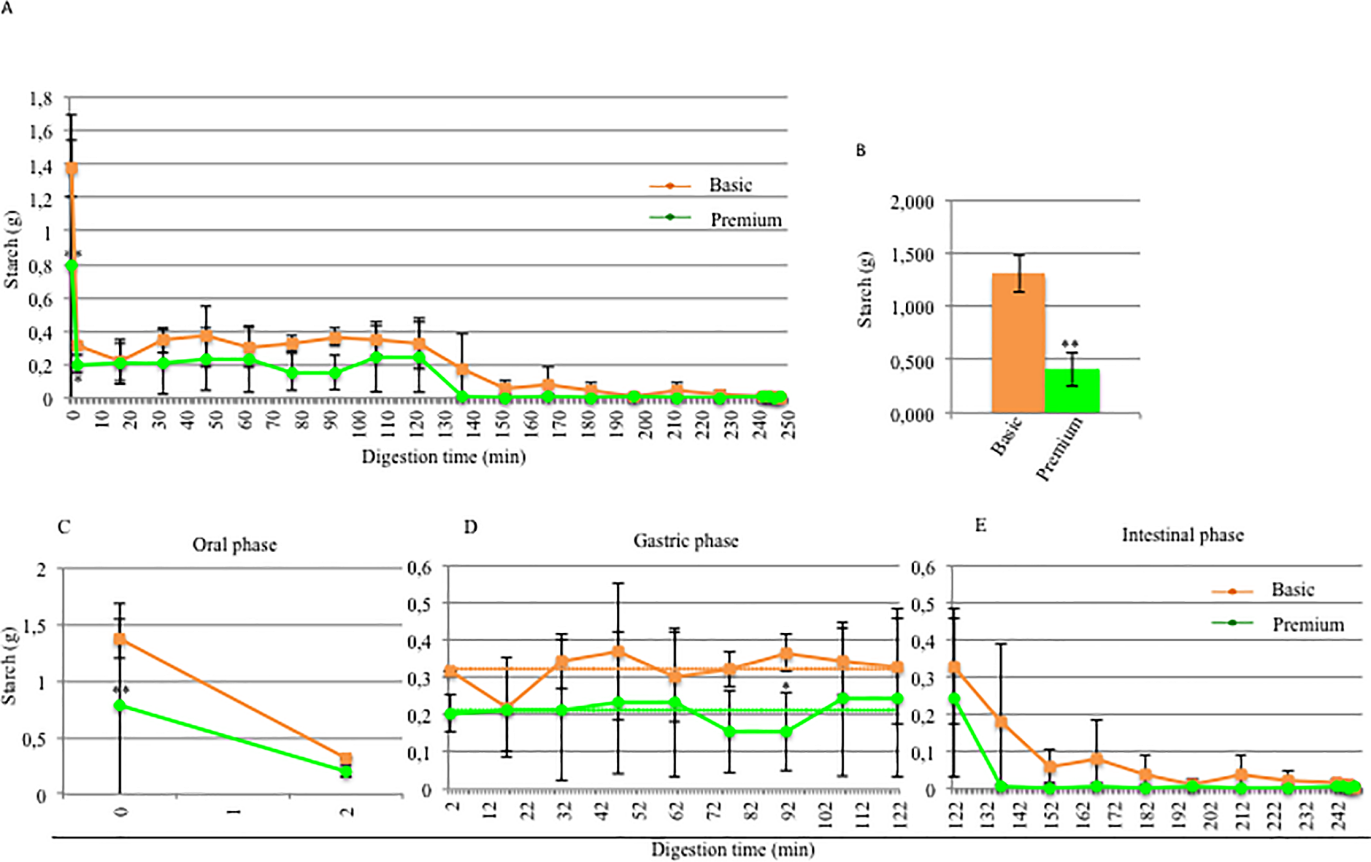
Starch digestion in the M.I.D.A. system of fermented or unfermented rice cream. A) Effect of fermentation on the starch content during the entire digestion process; B) comparison of the starch content at 0 min between the basic product (orange) and the premium product (green); starch content during oral (C), gastric (D) and intestinal phases (E) (orange: basic product; green: premium product). In D, the orange dotted line represents the mean starch content observed during the entire gastric phase for the basic product, while the green dotted line for the premium product. Each test was conducted in triplicate. Dots and squares represent the mean values, and bars represent the standard deviation. Student’s *t*-test * = *p*<0.05, ** = *p*<0.01.

In **Fig 5**an overall view of the digestion times (**A**), the starch level at T0 (**B**) and the separate digestion steps (**C, D, E**) are shown. Interestingly, basic and premium products had different starch contents at 0 min (Fig 5B) before the *in vitro* digestive process (1.309 g of starch for the basic product and 0.411 g for the premium product; this difference was statistically significant) suggesting that the fermentation by *Lactobacillu*s *paracasei* CBA L74 was able to reduce the starch content.

The different starch content during the oral phase (from 0 min to 2 min) in the rice cream-based foods (basic and premium) are reproduced in Fig 5C. After 2 min of incubation at 37°C with α-amylase, 0.317 g was found in the basic food (degree of starch conversion: 75.78%), while 0.202 g was found in the premium food (degree of starch conversion: 50,85%). The development of gastric starch digestion (from 2 min to 122 min) in the rice cream based-foods is shown in Fig 5D. During the entire gastric phase the starch content of the premium (mean starch content: 0.211 g) was less than the classical product (mean starch content: 0.324 g). Interestingly, in both cases, there was a fluctuating trend around the mean value during the gastric phase, suggesting, also in this case, that no digestion was occurring during this phase.

The evolution of the starch content both for basic and premium products during the last stage of *in vitro* digestion (from 122 min to 248 min) is described in Fig 5E. In the premium product, after the first 15 min of intestinal digestion, the starch was almost completely hydrolyzed as there was 0.007 g of starch corresponding to a degree of conversion of 98.29%; therefore, the starch content varied slightly during intestinal digestion, and the overall degree of conversion was fairly similar to that detected at 137 min (99.27%). Conversion of starch in the basic food was instead more gradual. After the first 15 min of the intestinal phase, there was 0.179 g of starch with a conversion degree of 86.32%; this continued to increase until the last minute of digestion reaching a final value of 99.69%. The final content of starch was similar in both products also in this case: at 248 min, there was 0.004 g in the basic product and 0.005 g in the premium product. It was possible to note complete digestion during the intestinal phase, but for the premium product the complete digestion of starch was sooner than in the case of the basic product.

### D-glucose analysis of rice cream foods with and without fermentation digested in the M.I.D.A. system

**Fig 6:**
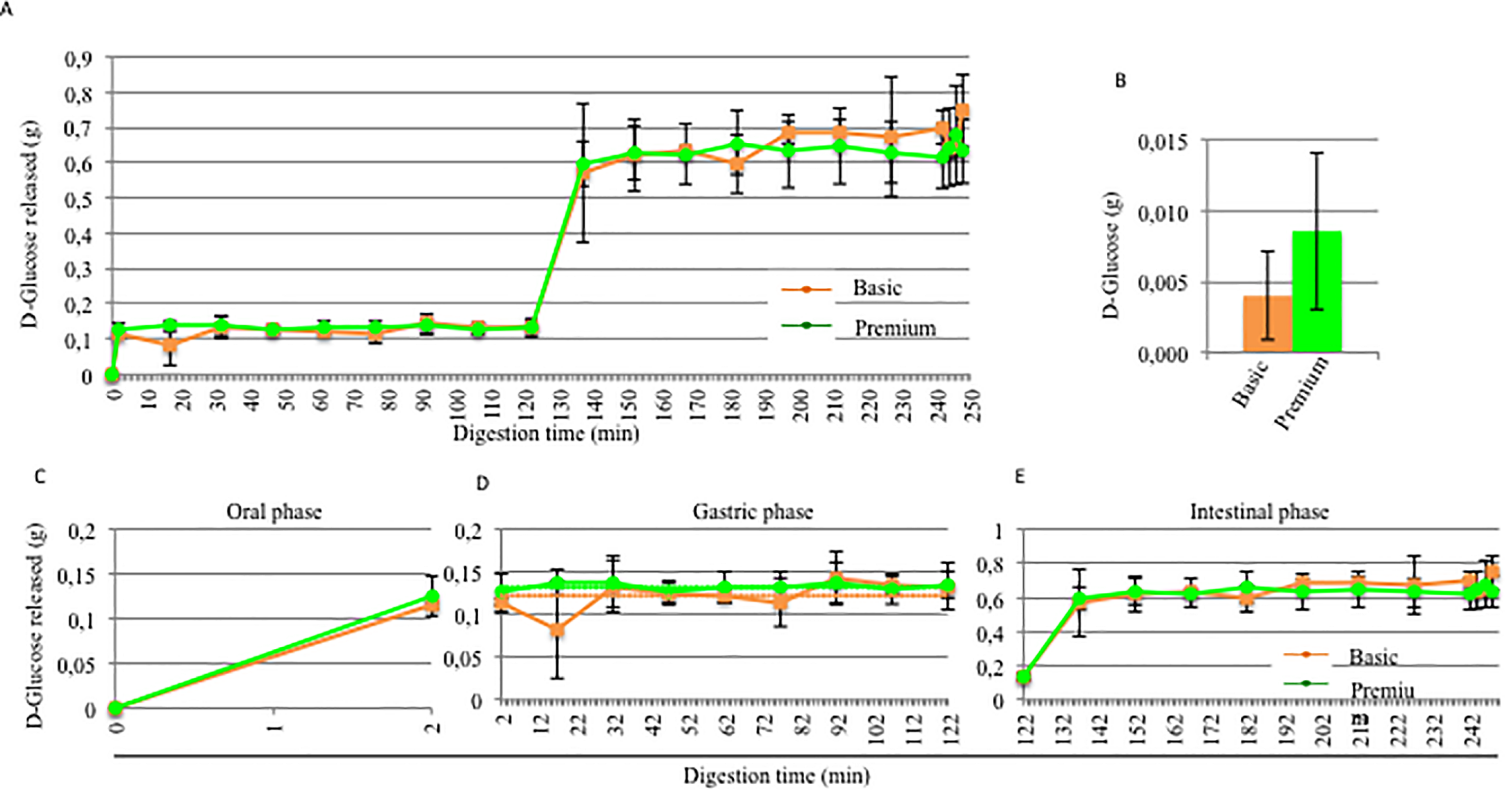
Effect of the fermentation process on the D-glucose released. A) Glucose released during the entire digestion process; B) D-glucose level at T0; Glucose released during the oral (C), gastric (D) and intestinal phases (E). (orange: basic product; green: premium product). In D, the orange dotted line represents the mean D-glucose content observed during the entire gastric phase for the basic product, while the green dotted line represents the mean D-glucose content for the premium product.

**Fig 7:**
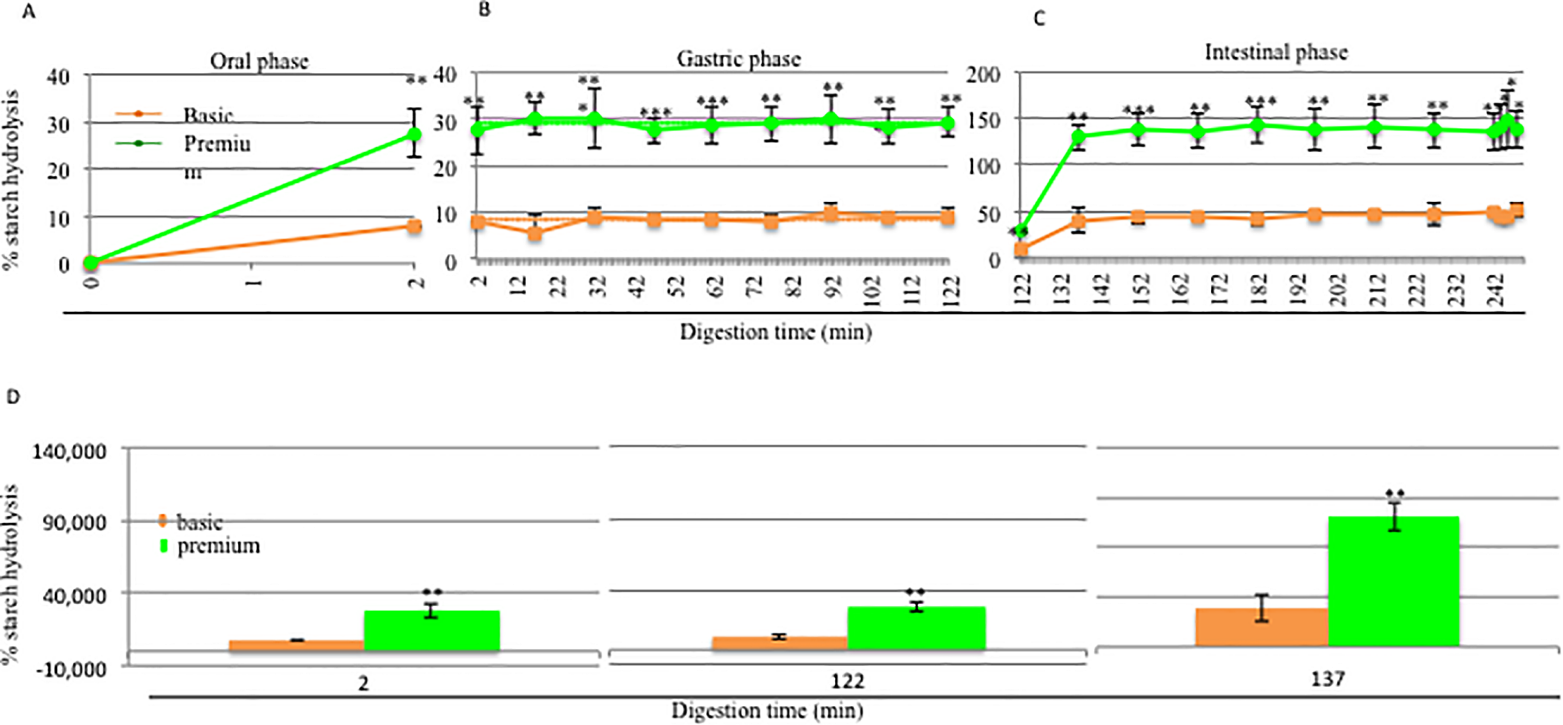
Effect of the fermentation process on the starch hydrolysis. Percentage of starch hydrolysis during the oral (A), gastric (B) and intestinal phases (C). (orange: basic product; green: premium product). (D) Focus on the differences (%) in the starch hydrolysis at different times. Percentage of starch hydrolysis between the basic (orange) and the premium (green) products at 2 minutes (oral), 122 minutes (intestinal), and 137 minutes (intestinal). Each test was conducted in triplicate. Dots and squares represent the mean values, and bars represent the standard deviation. Student’s *t*-test * = *p*<0.05, ** = *p*<0.01.

D-glucose produced through the enzymatic hydrolysis of starch in both the rice cream-based foods, basic and premium, was calculated using Eq 4, given in the methods section. In **Fig 6,** the D-glucose released during the entire digestion (**A**), the D-glucose present at T0 (**B**), and the D-glucose released during the separate steps (**C, D, E**) are shown. At time 0 (Fig 6B), the fermentation process appeared to affect the levels of glucose present at 0 min. In fact, in the basic product, there was a lower level of D-glucose (0.004 g), compared to the premium product (0.009 g). The D-glucose produced after 2 min of oral digestion in both the rice cream-based foods, basic and premium, is illustrated in Fig 6C. There was 0.115 g of D-glucose released in the oral sample from the basic product, while 0.126 g was found in the premium product after two minutes of digestion. D-glucose was released in only the oral (Fig 6C) and intestinal phases (Fig 6E), as expected. After the first 15 min of the intestinal phase, there was a similar level of D-glucose released: 0.570 g in the basic product and 0.590 g in the premium product. During the intestinal digestion, there were no significant changes in the amount of D-glucose released. At the end of the *in vitro* digestive process, 0.746 g was found in the basic and 0.632 g in the premium. Because of the significant difference in terms of starch observed at T0 between the basic and premium products, we studied the percentage of starch hydrolysis through the use of Eq 5, described in the methods section. In this case the study of the percentage of starch hydrolysis was more important because it could take into account of the initial starch content. **Fig 7**shows the percentage of starch hydrolysis during the oral (**A**), gastric (**B**) and intestinal steps (**C**) of digestion. The percentage of starch hydrolysis observed during the oral digestion for both of the rice cream-based foods is illustrated in Fig 7A: in the basic food, the percentage of starch hydrolysis was 7.89%, while in the premium product, it was 27.54%. The difference observed was statistically significant. The percentage of starch hydrolysis during gastric digestion for both rice cream-based foods is reproduced in Fig 7B. The mean percentage of starch hydrolysis to D-glucose was 8.39% for the basic product and 28.99% for the premium product.

The percentage of starch hydrolysis during the last stage of the *in vitro* digestion of the rice cream-based foods is reported in Fig 7C e 7D. After 15 min of intestinal digestion, the starch hydrolysis for the basic product was 39.13%, while the starch hydrolysis for the premium was 130.03%. At the end of the intestinal digestion (T248), a starch hydrolysis of 51.34% was found for the basic product and 138.40% for the premium product. All the percentage data points of the starch hydrolysis were statistically significant between the basic and premium products, confirming the better digestibility of the premium product.

## Discussion

“Tell me what you eat and I will tell you who you are” well summarize the importance of studying nutrition and digestion, in particular in the pediatric age. For this purpose, in vivo studies represent the best route to obtain informations, but only a few studies have been done in vivo on infants, because of ethical reasons and costs. For this reason few digestive dynamic systems have been described in the literature [7–8, 29]. These models consist of a sequence of bioreactors that reproduce physiological conditions in the different parts of the digestive tract. In order to reproduce as faithfully as possible the physiological reality of the gastrointestinal tract, several models of dynamic digestion in vitro have been developed [8]. Arnold and Dubois (1983) used a 19 mm inner diameter silicone tube and a peristaltic pump to reproduce gastric food mixing [30]. Molly et al. (1993) developed a multi compartment system consisting of six controlled reactors through computer simulating stomach, duodenum, jejunum, ileum, cecum, and colon [31]. Hoebler et al. (2002) developed a dynamic digestion system in which the pH and the pepsin content were automatically adjusted by pumps [32]. The main multi-compartment dynamic in vitro model that simulates the human gastrointestinal tract with the highest fidelity is the TIM-1 (TNO gatro-intestinal model) developed by TNO Nutrition and Food Research (Holland) and patented by Minekus et al. in 1996 [29]. The TIM consists of 4 serial compartments simulating stomach, duodenum, jejunum and ileum. TIM-1 was used to evaluate the bioavailability of folate in fortified milk [33], for the evaluation of the absorption of probiotics [34], for the evaluation of the release of phenolic compounds from food matrices [35]. It has also been used in the pharmaceutical field to evaluate digestion of drugs in various physiological conditions [36]. However, the model fails to fully reproduce fluid mechanics and mechanical forces in the gastro-intestinal tract [37]. An *ad hoc* system for child digestion was developed by Havenaar et al. [22], the TIMpediatric. This model simulates the conditions of the gastrointestinal tract specifically for infants, but was used exclusively for pharmaceutical purposes. As there are limited informations regarding infant digestion, we have described a new dynamic *in vitro* digestive system that is able to simulate the main stages of digestion, from oral to intestinal, of a six-month-old infant. The dynamics of our M.I.D.A system was guaranteed by the continuity of the simulated gastrointestinal tract, which was composed of three compartments, that were not considered as simply separate flasks but as districts interacting with each other. In each of these compartments, we simulated the physiologic temperature, pH and enzymatic profiles. Our model is, to the best of our knowledge, the only one resembling an infant gastrointestinal tract and that has been used for the purpose of studying the digestibility of nutrients, such as starch. Starch is the primary nutrient involved in energy intake since it is a source of D-glucose, which is the primary substrate for cell metabolism. The digestibility and absorption of starch has significant nutritional and physiological implications both in children and in adults. The extent of starch digestibility is influenced by the nature of the starch, food processing and the physiological status [38] and is generally not predictable in complex foods. Therefore we have used starch digestion to test our system.

The readout of starch digestion was the amount of residual starch, the D-glucose released and the percentage of starch hydrolysis in time. The first analysis was independent of the amount of starch loaded, whereas the other two were corrected for the initial amount loaded in the system. The M.I.D.A dynamic digestive system could digest starch no matter what sterilization method was used, in the correct compartment and allowed a punctual monitoring of the digestion process.

The next step was to challenge the M.I.D.A. system with a more complex food, specifically fermented or unfermented commercial rice cream. The rational of this experiment was to test the digestibility of the same very common baby food before and after the fermentation process carried out with a particular strain of *Lactobacillus* [10]. Fermentation is a metabolic process that releases energy from a sugar or other organic molecule, does not require oxygen or an electron transport system, and uses an organic molecule as the final electron acceptor. This process, applied to the food industry, is important for several reasons, such as the preservation of foods [39], the enhancement of the sensorial properties of food [40] or, more recently, the possibility to obtain functional foods [41].

In particular understanding the mechanisms of infant food digestion, fermented or unfermented, in the infant gastrointestinal tract is a key step for developing new foods with health benefits for the infant. In our experiments, the fermentation process alone was sufficient to reduce the starch amount in the rice cream confirming that fermentation is a process that helps food digestibility. Interestingly, during the intestinal phase of digestion, the percentage of starch hydrolysis in the premium product was significantly greater than the basic product. The percentage of starch hydrolysis is related to the starch present at time 0, consequentely taking into account the initial different amounts of the starch in the two foods tested. The starch content and the D-glucose released, instead, do not take into account the initial different amounts of the starch; this is why no statistically significant differences were observed between these two products in term of starch content and D-glucose released.

Increased starch digestibility in the premium food could be related to the amylolytic properties of the fermenting microflora that bring about the cleavage of amylase and amylopectin to maltose and D-glucose [42, 43]. Additionally, reduction in amylase inhibitor activity may be responsible for the increased starch digestibility [44, 45]. Reduction in phytic acid and other antinutrients due to fermentation [46] may also partly explain the improvement in starch and protein digestibility because phytic acid is known to inhibit amylolytic activity [47, 48]. Moreover, it was also demonstrated that fermentation causes proteolysis of the protein matrix surrounding the starch granules; therefore starch granules are released from protein matrix and are easily hydrolyzed by amylase, thus increasing the digestibility of starch [46].

In conclusion, the M.I.D.A. system was able to digest starch in the correct compartment and demonstrated that fermented baby food is better digested than the unfermented baby food.

Altough human clinical trials undoubtedly remain the best approach to study food digestion [6], they cannot always be conducted because of ethical reasons, technical constraints and costs. Our M.I.D.A. system represents an alternative method that is more similar to physiological reality that can be used to study digestion of not only starch or baby foods but also more complex meals. The proposed system could help to study and understand the nutrient decomposition, being an helpful tool to prevent the effect of nutrients excess (for example in the diabet disease) or deficiency. M.I.D.A. could let understand the effect of different food processings on food digestibility, also determining the interaction among different nutrients and other food components, and clarifying the effects on food digestion and absorption. In order to better understand food digestion regimes, it is crucial to control the transit and mixing times of bolus and chyme, also using automatic devices mimicking peristalsis and segmentation. In this way it is possible to determine the kinetics of food decomposition by correlating them to pathologies that modify the transit times of food. The nutrients thus obtained, suitably separated, can be brought into contact with intestinal cellular models, such as cell lines or intestinal organoids, in order to determine permeability and to predict the in-vivo absorption.

## Abbreviations

U/ml: Unit/milliliter
U/mg: Unit/milligram
AMG: Glucoamilase or amyloglucosidase
AGU/g: One amyloglucosidase unit/gram
U. Ph. Eur: Unit Pharmacopoeia Europaea
HTST: High Temperature Short Time
CFU: Colony Forming Unit
w/w: weight/weight
v/v: volume/volume
SDF: Simulated Digestive Fluid
SSF: Simulated Salivary Fluid
SGF: Simulated Gastric Fluid
SIF: Simulated Intestinal Fluid
GOPOD: Glucose oxidase/peroxidase
ABS: Acrylonitrile–Butediene–Styrene

## References

[1] Danaei G, Finucane M, Lu Y, Singh G, Cowan M, Paciorek C, et al (2011). National, regional, and global trends in fasting plasma glucose and diabetes prevalence since 1980: systematic analysis of health examination surveys and epidemiological studies with 370 country-years and 2·7 million participants. The Lancet 2011; 378: 31–40. 10.1016/S0140-6736(11)60679-X 21705069

[2] Lohi S, Mustalahti K, Kaukinen K, Laurila K, Collin P, Rissanen H, et al (2007). Increasing prevalence of coeliac disease over time. Aliment. Pharmacol. Ther. 2007; 26:1217–1225. doi: 10.1111/j.1365-2036.2007.03502.x 17944736 10.1111/j.1365-2036.2007.03502.x

[3] Eliasson AC (2004). Starch in food: Structure, function and applications. Woodhead Publishing.

[4] Hansen JT (2014). Netter's Clinical Anatomy. Elsevier—Health Sciences Division.

[5] Desai BB (2000). Chapter 19: Digestion, Absorption, Metabolism and Exctetion-Handbook of Nutrition and Diet, published by Taylor & Francis Inc.

[6] Deglaire A, Bos C, Tome D, Moughan PJ (2009). Ileal digestibility of dietary protein in the growing pig and adult human. British Journal of Nutrition 2009; 102(12), 1752–1759. doi: 10.1017/S0007114509991267 19706206 10.1017/S0007114509991267

[7] Ménard O, Cattenoz T, Guillemin H, Souchon I, Deglaire A, Dupont D, et al (2014). Validation of a new *in vitro* dynamic system to simulate infant digestion. Food Chemistry 2014; 145:1039–1045. doi: 10.1016/j.foodchem.2013.09.036 24128581 10.1016/j.foodchem.2013.09.036

[8] Guerra A, Etienne-Mesmin L, Livrelli V, Denis S, Blanquet-Diot S, Alric M (2012). Relevance and challenges in modelling human gastric and small intestinal digestion. Trend in Biotechnology 2012; 30(11):591–600. 10.1016/j.tibtech.2012.08.001 22974839

[9] Coles LT, Moughan PJ, Darragh AJ (2005). In vitro digestion and fermentation methods, including gas production techniques, as applied to nutritive evaluation of foods in the hindgut of humans and other simple-stomached animals. Animal Food Science and Technology:123–124, 421–444.

[10] Sarno M, Lania G, Cuomo ML, Nigro F, Passannanti F, Budelli A, et al (2014). Lactobacillus paracasei CBA L74 interferes with gliadin peptides entrance in Caco-2 cells. Int J Food Sci Nutr. 2014; 65(8):953–9. doi: 10.3109/09637486.2014.940283 25030417 10.3109/09637486.2014.940283

[11] Minekus M, Alminger M, Alvito P, Ballance S, Bohn T, Bourlieu C, et al (2014). A standardised static in vitro digestion method suitable for food–an international consensus. Food & Function 2014; 5:1113–1124. 24803111 10.1039/c3fo60702j

[12] Kaye J L (2011). Review of paediatric gastrointestinal physiology data relevant to oral drug delivery. Int. J. Clinic. Pharm. 2011; 33:20–24. 10.1007/s11096-010-9455-0 21365389

[13] Moules T, Ramsay J, Hendrick J (1998). The textbook of children’s nursing: part 1 2nd ed. Published by Nelson Thornes Ltd.

[14] Weaver LT, Austin S, Cole TJ (1991). Small intestinal length: a factor essential for gut adaptation. Gut 1991; 32(11):1321–3. 1752463 10.1136/gut.32.11.1321 PMC1379160

[15] Rossiter MA, Barrowman JA, Dand A, Wharton BA (1974). Amylase content of mixed saliva in children. Acta Pediatr. Scand. 1974; 63:389–92. 10.1111/j.1651-2227.1974.tb04815.x 4209508

[16] Seidel BM, Schubert S, Schulze B, Borte M (2001). Secretory IgA, free secretory component and IgD in saliva of newborn infants. Early Hum Dev. 2001; 62:159–64. 11282225 10.1016/s0378-3782(01)00130-x

[17] Lopez ME, Colloca ME, Paez RG, Schallmach JN, Koss MA, Chervonagura A (2003). Salivary characteristics of diabetic children. Braz Dent J. 2003;14:26–31. 12656461 10.1590/s0103-64402003000100005

[18] Bao J, Bergman CJ (2004). Chapter 9: The functionality of rice starch Starch in Food: Structure, Function and Application CRC Press Ed.

[19] Tavares AMP (2014). Investigating the effects of proteins on mycotoxins bioaccessibility and intestinal transport. Host: Egger C. National Health Institute, Dr. Jorge R, IP, Food and Nutrition Departmen, Lisbon, Portugal.

[20] Elashoff JD, Reedy TJ, Meyer JH (1982). Analysis of gastric emptying data. Gastroenterology 1982; 83(6):1306–1312. 7129034

[21] Hunt JN, Stubbs DF (1975). The volume and energy content of meals as determinants of gastric emptying. J Physiol. 1975; 245(1):209–25. 1127608 10.1113/jphysiol.1975.sp010841 PMC1330851

[22] Havenaar R, Anneveld B, Hanff LM, de Wildt SN, de Koning BA, Mooij MG, et al (2013). In vitro gastrointestinal model (TIM) with predictive power, even for infants and children? Int. J. Pharm. 2013; 457(1):327–32. doi: 10.1016/j.ijpharm.2013.07.053 23906665 10.1016/j.ijpharm.2013.07.053

[23] Patterson M, Rintala R, Lloyd DA (2000). A longitudinal study of electrogastrography in normal neonates. J Pediatr. Surg. 2000; 35:59–61. 10646775 10.1016/s0022-3468(00)80014-7

[24] Fallingborg J, Christensen LA, Ingeman-Nielsen M, Jacobsen BA, Abildgaard K, Rasmussen HH, et al (1990). Measurement of gastrointestinal pH and regional transit times in normal children. J. Pediatr. Gastroenterol. Nutr. 1990;11:211–4. 2395061 10.1097/00005176-199008000-00010

[25] Davies B, Morris T (1993). Physiological parameters in laboratory animals and humans. Pharm. Res. 1993; 10:1093–5. 8378254 10.1023/a:1018943613122

[26] Cystic Fibrosis Foundation, Borowitz D, Robinson KA, Rosenfeld M, Davis SD, Sabadosa KA, et al Cystic Fibrosis Foundation evidence-based guidelines for management of infants with cystic fibrosis. J Pediatr. 2009; 155(6 Suppl). 10.1016/j.jpeds.2009.09.001 PMC6324931 19914445

[27] Zoppi G, Andreotti F, et al (1972). Exocrine pancreas function in premature and full term neonates. Pediatr Res. 1972; 6:880–886. doi: 10.1203/00006450-197212000-00005 4678558 10.1203/00006450-197212000-00005

[28] Tamura M, Singh J, Kaur L, Ogawa Y (2015). Evaluation of digestibility of cooked rice grain using in vitro digestion technique. Agric. Eng. Int. 2015; 268–273.

[29] Minekus M, Marteaul P, Robert Havenaar R, Huis in’t Veld JHJ (1995). A Multicompartmental Dynamic Computer- controlled Model Simulating the Stomach and Small Intestine. ATLA 23 1995; 197–209.

[30] Arnold J, Dubois A (1983). In vitro studies of intragastric digestion. Digestive Disease and Sciences, 737–741. 10.1007/BF01312565 6872806

[31] Molly K., Woestyne M.V., & Vertsraete W. (1993). Devolpment of a five-step multichamber reactor as a simulation of the human intestinal microbial ecosystem. Applied microbiology and biotechnology, 254–258. 7763732 10.1007/BF00228615

[32] Hoebler C., & al. (2002). Development of an in vitro system simulating bucco-gastric digestion to assess the physical and chemical changes of food. Int. J. Food Sci. Nutr., 389–402. 10.1080/0963748021000044732 12396464

[33] Verwei M., & al. (2005). The effect of folate-binding proteins of folate from milk products. Trends Food Sci. Technol., 307–310

[34] Mattila-Sandholm, & al. (1999). Lactic acid bacteria with health claims-interactions and interference with gastrointestinal flora. Int. Dairy J., 25–35.

[35] Giz-Izquierdo, & al. (2002). An in vitro method to simulate phenolic compound release from the food matrix in the gastointestinal tract. European Food Research and Technology, 155–159.

[36] Blanquet S., & al. (2004). A dinamic artificial gastrointestinal system for studying the behavior of orally administred drug dosage forms under various physiological conditions. *Pharma res*. 10.1023/b:pham.0000022404.70478.4b 15139514

[37] Yoo J.Y., & Chen X.D. (2006). GIT physicochemical modeling-a critical review. Int J Food Engr.

[38] Niba LL (2005). Chapter 3: Carbohydrates: Starch–Handbook of Food Science, Technology, and Engineering– 4 Volume Set. Edited by Huy Y.H., published by Associate Editors, CRC Press.

[39] Ross RP, Morgan S, Hill C (2002). Preservation and fermentation: past, present and future. International Journal of Food Microbiology 2002; 79:3–16. 12382680 10.1016/s0168-1605(02)00174-5

[40] Pinto MF, Ponsano EHG, Franco BDGM, Shimokomaki M (2002). Charqui meats as fermented meat products: role of bacteria for some sensorial properties development. Meat Science 2002; 61:187–191. 22064008 10.1016/s0309-1740(01)00184-x

[41] Zagato E, Mileti E, Massimiliano L, Fasano F, Budelli A, Penna G, et al (2014). Lactobacillus paracasei CBA L74 metabolic products and fermented milk for infant formula have anti-inflammatory activity on dendritic cells in vitro and protective effects against colitis and an enteric pathogen in vivo. PLoS One 2014 10;9(2). 10.1371/journal.pone.0087615 PMC3919712 24520333

[42] Sindhu SC, Khetarpaul N (2001). Probiotic Fermentation of Indigenous Food Mixture: Effect on Antinutrients and Digestibility of Starch and Protein. Journal of Food Composition and Analysis 2001; 14:601–609.

[43] Bernfeld P (1962). In “Comparative Biochemistry” (Florkin M. and Mason H. W., Eds.), Vol. III, p. 355 Academic Press, New York.

[44] Sharma A, Kapoor AC (1996a). Effect of various types of fermentation on in vitro protein and starch digestibility of differently processed pearl millet. Nahrung Food 1996; 40(3):142–145. 8766667 10.1002/food.19960400309

[45] Sharma A, Kapoor AC (1996b). Levels of antinutritional factors in pearl millet as affected by processing treatments and various types of fermentation. Pl. Foods Hum. Nutr. 1996; 49(3):241–252. 10.1007/BF01093221 8865334

[46] Knuckles BE, Kuzmicky DD, Betschart AA (1985). Effect of phytate and partially hydrolyzed phytate on in vitro protein digestibility. J. Food Sci. 1985; 50:1080–1082.

[47] Thompson LU, Yoon JH (1984). Starch digestibility as affected by polyphenol and phytic acid. J. Food Sci.1985; 49:1228–1229.

[48] Singh J, Dartois A, Kaur L (2010). Starch digestibility in food matrix: a review. Trends Food Sci. Technol. 2010; 21(4):168–180.

